# Body mass index, waist-hip ratio and risk of chronic medical condition in the elderly population: results from the Well-being of the Singapore Elderly (WiSE) Study

**DOI:** 10.1186/s12877-016-0297-z

**Published:** 2016-06-18

**Authors:** Restria Fauziana, Anitha Jeyagurunathan, Edimansyah Abdin, Janhavi Vaingankar, Vathsala Sagayadevan, Saleha Shafie, Rajeswari Sambasivam, Siow Ann Chong, Mythily Subramaniam

**Affiliations:** Research Division, Institute of Mental Health, Buangkok Green Medical Park, 10 Buangkok View, Singapore, 539747 Singapore

**Keywords:** Body mass index, waist-hip ratio, chronic medical conditions, elderly

## Abstract

**Background:**

The aim of the current study was to establish the prevalence and relationship of Body Mass Index (BMI) and Waist-Hip Ratio (WHR) with chronic health conditions and their associated socio-demographic correlates in the elderly population of Singapore.

**Methods:**

The data was extracted from the Well-being of the Singapore Elderly (WiSE) study, a comprehensive single phase, cross-sectional, population-based, epidemiological study conducted in 2013 among Singaporean residents (*n* = 2565) aged 60 years and above with a mean age of 72.7 years (range 60 to 105, SD = 9.53). The respondents were assessed with anthropometric measurements including height, weight, BMI, waist circumference, hip circumference and WHR. Participants provided information on their socio-demographic details and chronic health conditions.

**Results:**

Prevalence of those who were obese, overweight, normal and underweight based on BMI was 8.7 %, 33.4 %, 52.5 % and 5.5 % respectively. Malays were more likely to be overweight compared to Chinese and Indians, while Malays and Indians were more likely to be obese compared to Chinese. Participants who were never married were less likely to be overweight compared to married. Participants aged 85 years and above were more likely to be underweight compared to those aged 60-75 years. Prevalence of high WHR (above 0.90 for men and 0.80 for women) was 79.8 % and this was more pervasive amongst Indians. Participants who were homemakers were more likely to have high WHR while those with tertiary education tended to have low WHR. Being overweight was associated with hypertension and heart problems, while obesity was associated with hypertension and diabetes, and a high WHR was associated with hypertension and diabetes. There were no significant differences in the other chronic conditions in this elderly population.

**Conclusions:**

This study demonstrates the importance of anthropometric measurements in the elderly and its association with certain chronic physical conditions, indicating their utility in the clinical management of these conditions in the elderly.

**Electronic supplementary material:**

The online version of this article (doi:10.1186/s12877-016-0297-z) contains supplementary material, which is available to authorized users.

## Background

Statistics around the world have shown that the proportion of the elderly has been steadily increasing over the last decade [1]. Singapore is a multi-ethnic society comprising mainly Chinese (74.3 %), Malays (13.3 %), Indians (9.1 %) and Other ethnic groups (3.3 %) [2]. Singapore has one of the world’s fastest ageing population [3]. As of September 2014, the Ministry of Manpower and Singapore Department of Statistics reported that 11.2 % of the population comprised citizens over 65 years compared to 7.8 % in 2004.

According to the National Population and Talent Division of Singapore [4], the life expectancy of Singaporeans has increased by 10 years over the last three decades, with the average life expectancy reported as 82 years in 2010. Several changes in the body take place as one ages, namely physiological ageing, which affects body mass and composition even in the absence of diseases [5]. The ageing process involves physiological and nutritional changes that are manifested by height, weight and muscular mass loss and an increase in fat mass, including adipose tissue redistribution, with fat accumulation in the trunk and viscera [6–8]. The aging process also results in poor energy regulation, reduced hormonal levels and changes in the metabolic rate, which in turn affect their anthropometric measurements and risk of cardiovascular diseases [9].

Anthropometry is an essential tool in geriatric nutritional assessment and is used to evaluate weight related conditions (underweight to obese) among the elderly. It provides an indirect evaluation of body composition [10] and risk of acute and chronic diseases [11]. A standard anthropometric measurement tool employed by the World Health Organization (WHO) is the Body Mass Index (BMI) which uses a ratio of height and weight to classify adults as underweight, normal, overweight or obese [12]. The measure is an indicator for a number of health risks such as high blood pressure, diabetes, heart diseases as well as a gauge of nutritional deficiency [13]. While widely used and deemed as the easiest assessment of a healthy weight, it fails to take into account age, gender or body shape and composition [14]. The categories are primarily based on evidence from studies of morbidity and mortality in younger adults. Woo et al [15] demonstrated that BMI was not a sensitive index to measure obesity or under-nutrition as it is unable to recognize a high fat percentage, a low standardized lean mass value and does not account for an increase in muscle or excess fat distribution. Other anthropometric measurements such as waist circumference and waist-hip ratio (WHR) are regarded as alternatives to BMI. As fat in the abdominal region is associated with increased health risks, the National Institute for Health and Clinical Excellence (NICE) and National Institute of Health (NIH) recommends these measures as a practical tool to measure risk factors for diseases like diabetes and hypertension, especially in persons with a BMI range under 35 kg/m^2^ [16, 17]. Several studies have shown that there is a strong positive association between cardiovascular risk factors with measures of waist circumference or WHR instead of BMI alone [18–21].

This article aims to (i) establish the prevalence of obesity, as well as underweight in the elderly population, (ii) explore the socio-demographic factors which predict the anthropometric characteristics in the elderly and finally, (iii) explore the relationship between BMI and WHR with chronic health conditions in an elderly Asian population.

## Methods

### Sample

The study used data from the Well-being of the Singapore Elderly (WiSE) study, a comprehensive single phase, cross-sectional population-based epidemiological survey. Participants in the study were Singapore residents including Singaporean Citizens and Permanent Residents aged 60 years and above (mean 72.7, SD = 9.53, range 60 to 105) who were living in Singapore at the time of the survey. The study also included participants who were in day care centres, nursing homes and institutions during the course of the study. Residents not living in Singapore during the period of the study were excluded. Participants were randomly selected from an administrative database of residents in Singapore. A total of 2565 participants were recruited and disproportionate random sampling was used to ensure the appropriate proportions of the three main ethnic groups. This sample’s ethnic distribution was Chinese (*n* = 1012, 39.5 %), Malay (*n* = 745, 29.0 %), Indian (*n* = 772, 30.1 %) and Other ethnicities (*n* = 36, 1.4 %).

BMI and WHR were measured in 2285 and 2327 participants respectively. Missing data resulted from participants who were uncomfortable with the process, refused the physical measurements or were wheelchair bound or bedridden. Thus, interviewers were unable to take the measurements for these participants.

The study was approved by the institutional ethics review boards of participating institutions (National Healthcare Group Domain Specific Review Board (DSRB) as well as the SingHealth Centralised Institutional Review Board (CIRB)) and all participants gave written informed consent. In the event that participants were unable to do so, a written informed consent was taken from their legally acceptable representative before including the respondent in the study.

### Data collection

The survey was conducted from October 2012 to December 2013. All participants in the study received a full extensive assessment, lasting approximately 2–3 hours that involved the collection of data such as age (60-74, 75-84, 85+), gender (men, women), ethnicity (Chinese, Malay, Indian, Others), marital status (never married, married/cohabiting, widowed, divorced/separated), education (none, did not complete primary, completed primary, completed secondary, completed tertiary), employment status (paid work, unemployed, homemaker, retired), living circumstances, socio-economic status, medical history – namely hypertension, heart problems, diabetes, transient ischemic attacks (TIAs) and stroke – and a physical and neurological examination which is based on NEUROEX (a brief fully structured neurological assessment with objectified quantifiable measures of anthropometric measurements) [22].

The investigators were trained by a senior researcher on the use of the instruments and on conducting a proper physical and neurological examination. This core team of investigators then conducted training for the field interviewers hired by a contract survey firm. Further details on the WiSE methodology can be found in an earlier article [23].

### Measures

#### Anthropometric measurements

Height and weight were measured by trained field interviewers with participants wearing indoor clothing without shoes, using a tape measure and digital standing scale which was calibrated before every use. Additionally, to minimise measurement or reporting biases, the core team of investigators conducted field observations and monthly debriefs to identify problem areas and the need for re-training or revision of processes.

#### Body Mass Index (BMI)

BMI is defined as the weight in kilograms divided by the square of the height in meters (kg/m^2^). Cut-off standards by the World Health Organization (WHO) were used [12]. A BMI of less than 18.5 was classified as underweight, BMI of 18.5 to 24.9 as normal weight, BMI of ≥25.0 up to 30.0 was classified as overweight and BMI of ≥30.0 was classified as obese.

#### Waist circumference

The circumferences were measured to the nearest centimetre using a flexible tape with the respondent standing. In women, the abdominal circumference (waist) was measured as the narrowest part of the body between chest and hips and in men it was measured at the level of the umbilicus.

#### Hip circumference

The participant’s hip circumference was measured as the maximum circumference around the buttocks posteriorly at the level of greater trochanters (hip bones) and measured in centimetres.

#### Waist to Hip Ratio (WHR)

WHR was determined by dividing waist circumference by hip circumference. WHO [24] recommends cut-off points for waist circumference as 85 cm and 80 cm and WHR cut-off values of 0.90 and 0.80 for men and women respectively, where a higher ratio indicates an increased risk of various health complications.

#### Statistical analyses

All statistical analyses were performed using the Statistical Analysis Software (SAS) System version 9.2. All estimates were weighted to ensure that the survey findings were representative of the Singapore elderly population in 2011.

Descriptive analyses were performed to establish the weighted proportions in each BMI category and waist to hip ratio among the socio-demographic variables. To examine the associations between BMI categories, waist to hip ratio and socio-demographic variables, chi-square (χ^2^) tests were used in the bivariate analysis followed by multinomial and multiple logistic regressions in multivariate analysis. A series of logistic regression models were also used to examine the associations between BMI categories and waist to hip ratio with chronic health conditions. Age group, ethnicity, gender, marital status, education and employment status were included as socio-demographic correlates of BMI and waist to hip ratio in the analysis. Possible interactions between independent variables were also tested by including interaction terms in the model. If the term was significant, the interaction between the variables was included in the final model.

Given that there were a number of missing values in dependent [BMI (*n* = 276) and waist to hip ratio (*n* = 238)] and independent variables [marital (*n* = 2), education (*n* = 15) and employment status (*n* = 31)], there was a possibility that the regression analysis based on complete case analysis may introduce bias if the likelihood of missing data is associated with independent variables. We examined the missing data mechanisms using multiple logistic regression analysis and found that the data were related to age, ethnicity and employment status, suggesting missing not at random (MNAR) (Additional file 1: Table S1). To handle missing values, we employed multiple imputation by chained equations (MICE) to generate 20 complete data sets. The built-in predictive mean matching and multinomial logistic regression analyses in MICE model were utilized to replace each missing values for continuous and categorical variables [25]. The separate estimates and standard errors of each of the 20 data sets were pooled according to Rubin’s rules using multiple imputation estimates module in STATA version 13.1 (Stata Corp). Statistical significance was evaluated at the 0.05 level using 2-sided tests.

## Results

A total of 2565 participants completed the study, yielding a response rate of 66 %. The overall prevalence of obesity and overweight was 8.7 % and 33.4 % respectively while underweight was 5.5 %. The prevalence of normal BMI was 52.5 %. Table 1 shows the weighted proportions of BMI and WHR category in the study sample as well as among the different socio-demographic groups.

**Table 1:** Socio-demographic characteristics of sample by BMI category and WHR category

Variable	Category	Sample			BMI Category %				Waist-Hip Ratio %	
		Unweighted N (*N* = 2565)	Unadjusted^a^ %	Adjusted^b^ %	Underweight (*n* = 146)	Normal weight (*n* = 1061)	Overweight (*n* = 759)	Obese (*n* = 319)	Low WHR (*n* = 401)	High WHR (*n* = 1926)
Age Group (in years)	60–74	1494	58.2	75	3.7	51.2	35.5	9.5	20.6	79.4
	75–84	669	26.1	19.5	10.2	56.2	27.1	6.4	19.1	80.9
	85+	402	15.7	5.5	17.3	59.9	19.8	2.9	16.5	83.5
Ethnicity	Chinese	1012	39.5	83.3	5.7	55.4	32.7	6.2	21.1	78.9
	Malay	745	29	9.3	5.5	36.3	37.4	20.3	16.3	83.7
	Indian	772	30.1	6	3.2	39.9	35.8	21.1	13	86.9
	Others	36	1.4	1.4	2.9	33.5	38.9	24.7	19.2	80.8
Gender	Men	1117	43.5	44.1	5.4	54.1	34.1	6.4	20.9	79.01
	Women	1448	56.5	55.9	5.5	51.2	32.8	10.5	19.5	80.5
Marital Status	Never Married	136	5.3	8	5.6	63.2	23.6	7.6	25.9	74.1
	Married/Cohabiting	1484	57.9	64	4.6	50.9	35.6	8.9	19.4	80.6
	Widowed	836	32.6	22.5	8.4	51.7	31.3	8.6	18.7	81.3
	Divorced/Separated	107	4.2	5.5	2.8	59.5	29.2	8.1	25.5	74.5
Education	None	511	20	16.5	8.2	48.6	34.1	9.03	17.1	82.9
	Some, but did not complete primary	620	24.3	23.9	6.6	50.9	34.2	8.3	16.1	83.9
	Completed primary	640	25.1	24.8	5.7	53.8	31.04	9.4	21.3	78.7
	Completed secondary	517	20.3	22.4	4.3	51.1	35.1	9.5	19.8	80.2
	Completed tertiary	262	10.3	12.4	1.9	60.2	31.9	6.03	30.3	69.7
Employment Status	Paid work (part-time and full-time)	688	27.2	33.9	3.2	55.6	34.1	7.1	24.02	75.9
	Unemployed	32	1.3	1.5	2.5	57.8	36.8	3.02	29.03	70.9
	Homemaker	808	31.9	26.3	5.9	45.5	35.8	12.7	14.5	85.5
	Retired	1006	39.7	38.3	7.3	54.3	30.7	7.7	20.1	79.9

^a^ Adjusted figures refer to weighted estimates after adjusting for survey weights to ensure that the survey findings were representative of the Singapore elderly population in 2011.

^b^ Unadjusted figures refer to estimates from the sample without adjustment for survey weights.

Multinomial logistic regression analyses revealed that participants aged 75 years and above were less likely to be obese than those aged 60 – 74 years. A comparison among the ethnic groups showed that Malays were more likely to be overweight and Indians were more likely to be obese as compared to the Chinese. Participants who were never married or were single were less likely to be overweight compared to those who were married. Participants above the age of 85 years were more likely to be underweight than those who were 60 – 74 years old (Table 2).

**Table 2:** Prevalence (95 % CI) of BMI category by socio-demographic characteristics

Variable	BMI								
	Underweight			Overweight			Obese		
	Odds Ratio	(95 % CI)	P Value	Odds Ratio	(95 % CI)	P Value	Odds Ratio	(95 % CI)	P Value
Age Group (in years)									
85+	2.5	(1.2,5.2)	0.02	0.6	(0.4,1.0)	0.065	0.2	(0.1,0.5)	<0.000
75–84	1.8	(0.9,3.4)	0.074	0.9	(0.6,1.2)	0.371	0.4	(0.3,0.7)	<0.000
60–74	1 (reference)			1 (reference)			1 (reference)		
Ethnicity									
Malay	1.3	(0.8,2.1)	0.235	1.4	(1.1,1.9)	0.01	3.2	(2.3,4.3)	<0.000
Indian	0.8	(0.5,1.4)	0.496	1.9	(0.9,1.7)	0.058	3.3	(2.4,4.4)	<0.000
Others	1.2	(0.1,11.6)	0.871	1.7	(0.6,4.7)	0.348	5.9	(2.0,17.0)	0.001
Chinese	1 (reference)			1 (reference)			1 (reference)		
Gender									
Women	0.8	(0.4,1.6)	0.482	0.8	(0.6,1.2)	0.361	1.1	(0.7,1.8)	0.544
Men	1 (reference)			1 (reference)			1 (reference)		
Marital Status									
Never Married	0.94	(0.3,2.7)	0.235	0.4	(0.3,0.8)	0.004	0.7	(0.3,1.3)	0.213
Widowed	1.1	(0.5,2.2)	0.881	0.9	(0.7,1.4)	0.884	0.8	(0.5,1.3)	0.451
Divorced/Separated	0.6	(0.1,2.9)	0.501	0.7	(0.4,1.4)	0.315	0.7	(0.3,1.5)	0.31
Married/Cohabiting	1 (reference)								
Education									
None	2.6	(0.7,9.5)	0.138	0.6	(0.3,1.1)	0.087	1.3	(0.7,2.7)	0.425
Some, but did not complete primary	2.7	(0.8,8.6)	0.126	0.8	(0.5,1.3)	0.341	1.4	(0.7,2.6)	0.308
Completed primary	2.4	(0.7,7.9)	0.17	0.7	(0.4,1.1)	0.16	1	(0.5,1.9)	0.979
Completed secondary	1.8	(0.8,3.9)	0.203	1	(0.6,1.7)	0.971	1.2	(0.6,2.2)	0.585
Completed tertiary	1 (reference)			1 (reference)			1 (reference)		
Employment Status									
Unemployed	1.1	(0.2,6.9)	0.93	2.6	(0.7,9.8)	0.158	1.6	(0.3,7.9)	0.534
Homemaker	1.8	(0.7,4.6)	0.239	1.4	(0.9,2.2)	0.156	1.7	(1.0,2.9)	0.057
Retired	1.8	(0.8,3.9)	0.15	1.1	(0.8,1.6)	0.447	1.2	(0.8,1.8)	0.406
Paid work (part-time and full-time)	1 (reference)			1 (reference)			1 (reference)		

WHR across the socio-demographic groups is shown in Table 3. The prevalence of high WHR in our sample was 79.8 % (*n* = 1926). Multiple logistic regressions showed that participants who were Indians were more likely to have high WHR as compared to participants of Chinese ethnicity. Homemakers were more likely to have high WHR than those who were employed. Participants with tertiary education or higher were more likely to have lower WHR.

**Table 3:** Prevalence (95 % CI) of High WHR category by socio-demographic characteristics

Variable	High WHR		
	Odds Ratio	(95 % CI)	P Value
Age Group (in years)			
75–84	0.8	(0.6,1.3)	0.479
85+	1.2	(0.7,2.1)	0.48
60–74	1 (reference)		
Ethnicity			
Malay	1.2	(0.9,1.6)	0.217
Indian	1.9	(1.4,2.5)	<.0001
Others	1.4	(0.5,3.4)	0.512
Chinese	1 (reference)		
Gender			
Women	0.8	(0.6,1.2)	0.367
Men	1 (reference)		
Marital Status			
Never Married	0.8	(0.5,1.5)	0.525
Widowed	0.9	(0.6,1.3)	0.547
Divorced/Separated	0.8	(0.4,1.4)	0.418
Married/Cohabiting	1 (reference)		
Education			
None	2.1	(1.1,3.8)	0.016
Some, but did not complete primary	2.2	(1.3,3.6)	0.002
Completed primary	1.6	(0.9,2.6)	0.051
Completed secondary	1.8	(1.1,2.9)	0.024
Completed tertiary	1 (reference)		
Employment Status			
Unemployed	0.8	(0.3,2.3)	0.627
Homemaker	1.9	(1.1,3.0)	0.011
Retired	1.30	(0.9,1.9)	0.14
Paid work (part-time and full-time)	1 (reference)		

In multiple logistic regression, we found that those who were overweight were more likely to have hypertension (*p* = <0.000) and heart conditions (*p* = 0.017), while those who were obese were more likely to have hypertension (*p* = <0.000) and diabetes (*p* = 0.021). Those with a higher WHR were more likely to have hypertension and diabetes (*p* = 0.003 and *p* = 0.002 respectively). Other chronic conditions like stroke and TIAs were not significantly related to BMI and WHR (Table 4). There were no significant interactions found between the independent variables.

**Table 4:** Prevalence (95 % CI) of Chronic Health Conditions by BMI and WHR characteristics

Chronic Health Conditions	BMI									High WHR		
	Underweight			Overweight			Obese					
	Odds Ratio	(95 % CI)	P Value	Odds Ratio	(95 % CI)	P Value	Odds Ratio	(95 % CI)	P Value	Odds Ratio	(95 % CI)	P Value
	1(reference)									1(reference)		
Hypertension	0.7	(0.4,1.2)	0.204	1.8	(1.3,2.6)	<0.000	2.2	(1.4,3.3)	<0.000	1.6	(1.2,2.3)	0.003
	1(reference)									1(reference)		
Heart Problems	0.9	(0.4,1.9)	0.774	1.7	(1.1,2.5)	0.017	1.4	(0.8,2.3)	0.194	1.3	(0.8,2.1)	0.248
	1(reference)									1(reference)		
Diabetes	0.7	(0.4,1.5)	0.385	1.4	(1.0,1.9)	0.058	1.5	(1.1,2.2)	0.021	1.9	(1.3,2.7)	0.002
	1(reference)									1(reference)		
TIAs^a^	0.6	(0.1,3.0)	0.523	1.2	(0.4,3.4)	0.777	2.4	(0.8,7.2)	0.124	1.4	(0.4,4.6)	0.579
	1(reference)									1(reference)		
Stroke	1.3	(0.5,3.0)	0.591	1.1	(0.7,2.0)	0.639	1.1	(0.6,2.2)	0.683	1.7	(0.9,3.2)	0.101

^a^ Transient Ischemic Attacks

## Discussion

Our analyses indicated that 8.7 % of our elderly population were obese and 33.4 % were overweight. Those of Indian or Malay ethnicity and aged between 60 – 74 years were more likely to have high BMI. The Singapore National Health Study (NHS) in 2010 [26] reported that 7.2 % of those aged between 60 and 69 years were obese. The difference in prevalence could be due to the different age range of the population of focus (aged 18–65 years) in the NHS sample. A survey done in the US which examined the prevalence of obesity showed that prevalence of obesity was lower among those aged above 75 years as compared to those aged 65 – 74 years [27]; a trend similar to our population. This trend was also observed in populations in Spain, Italy, as well as Germany [28–30]. Body mass progressively declines with age from the reduction of lean body-fat mass, which accounts for the lower occurrence of obesity in the later years [31, 32].

The present study found that 5.5 % of participants sampled were underweight which was significantly associated with the oldest old (i.e. 85 years and above). Comparable results were found in Spain, where those above the age of 80 years were more likely to be underweight than those in the younger age groups [30]. A decrease in metabolic rate, loss of lean body mass and changes in the sense of taste and smell are the results of physiological aging, which in turn, causes unintentional weight loss [33]. Combined with physiological changes in the body that follow with natural ageing, the occurrence of multiple medical problems and other physical or cognitive impairments, this weight loss is inevitable [30]. While weight loss is a normal part of the aging process, losing 5–10 % of body weight over a period of 1–12 months might be indicative or predictive of pathology in the elderly [31]. One of the worrying issues with weight loss in the elderly, and especially the oldest old, is the risk of under-nutrition. The National Diet and Nutrition Survey among an elderly population in the United Kingdom (>65 years) revealed that 14 % were at a medium or high risk of under-nutrition based on a measure of low BMI and recently reported weight loss [32].

Studies have shown associations between marital status and becoming overweight and obese. A study by Tzotzas et al, [34] revealed that those who were married, divorced and separated had a higher risk of being overweight and obese compared to those who were never married. Single individuals tend to have the lowest prevalence of overweight and obesity [35] as they pay more attention to their diet, physical activity and overall well-being [36]. A survey conducted in Britain found that married couples often fail to meet the recommended 150 minutes of physical activity compared to single men and women [37], which could explain the elevated risks in this group.

Numerous studies in different cultures have reported that persons in the obese or overweight BMI range were at a greater risk of developing hypertension, diabetes and heart diseases [38–40]. Results of the current study also reflected these results. In a multi-ethnic, multi-national research, BMI was found to have a strong association with hypertension regardless of age and sex [41]. Our findings also showed that BMI was not a significant risk factor for other chronic illnesses like stroke, and TIAs. This suggests that a single anthropometric measurement may be inadequate and that a panel of measurements may provide a more predictive risk assessment of medical conditions in the elderly.

A high WHR was observed in 79.8 % of our sample. Risk factors of higher WHR were lower education, homemakers and Indian ethnicity. The National Health and Nutrition Examination Survey data indicates that waist circumference increases with age, with the increase being larger in older adults up to 70 years of age [42]. Starting from middle age till the years of 80 and above, subcutaneous fat decreases and fat is redistributed from the subcutaneous to the visceral depots [43] which results in an increase in WHR.

The current study found that those with at least tertiary education were found to have a lower WHR. A Swedish study done among women aged 45 – 73 years showed that weight increases (in tandem with WHR) throughout the lifetime of those who had lower educational attainment [44]. Conversely, a study by Rosmond and Bjorntorp [45] showed an association of high WHR with low education. Cutler and Lleras-Muney [46] found that highly educated individuals were more likely to exercise as they were more likely to possess knowledge and awareness of health risks, allowing them to develop healthier behaviours [47]. In a previous study in Singapore, it was shown that there was an increase in the odds ratio of high blood pressure, cholesterol levels and fasting blood glucose levels amongst those with high WHR [48]. The current study, along with others demonstrates a significant the association between diabetes, hypertension and high WHR, highlighting the validity of WHR as a strong measure of risk for diabetes and hypertension [21, 49–52].

When comparing the differences between ethnicity, BMI and WHR, a study done by Deurenberg-Yap et al [53] amongst the Singaporean population showed that the prevalence of obesity was highest in Malays followed by Indians and Chinese. Similarly, our results show that BMI and WHR varied between ethnicities. A higher prevalence of overweight and obesity was found among Malays while Indians showed a high prevalence of obesity and WHR. Studies done in Singaporean and Malaysian population have shown that certain ethnic groups (i.e. Indians and Malay) were more likely to have higher BMI [54–56] as well as WHR [53]. These variances between ethnic groups could be due to different dietary preferences, lifestyle, physiological differences (i.e. differences in trunk-to-leg-length ratio and differences in slenderness) as well as differences in genetic backgrounds [57–59]. For example, Asian Indians have been found to have higher abdominal fat compared to Europeans with the same BMI, resulting in a higher WHR [60]. The Singapore NHS reported similar results where the prevalence of obesity was highest in the Malays, followed by Indians and Chinese. Very little emphasis is placed on anthropometric measurements and its risk on health in the elderly population. Reduction in muscle mass is an important determinant of physical function and metabolic rate and leads to the clinical hazards of obesity appearing at a lower BMI in the elderly [61]. The decrease in BMI occurs at the expense of losing muscle mass and an increase in WHR. This phenomenon mainly occurs due to changes in total adiposity and in body weight. The elderly population also faces gradual bone loss in the femur and radius throughout their ageing [62], which makes it difficult to assign an accurate cut-off for anthropometric measurements.

There are a few limitations to our study. Firstly, the majority of study participants were recruited from their homes, so the access to nursing home residents for recruitment was limited. Secondly, some of the participants were non-ambulant which precluded measurements of BMI and WHR. And lastly, the study has a cross-sectional design, so it limits any causal inferences.

These limitations notwithstanding, this is a nationwide population-based survey, representative of a multi-ethnic elderly population in Singapore with a large sample size. The study was a single phase assessment, using widely accepted assessments and questionnaires with face-to-face interviews and using objective measurements instead of self-reports, which ensured accurate and detailed collection of information from all individuals.

## Conclusions

The inclusion of WHR measurements in addition to BMI provides a more comprehensive and accurate association of risk factors in an elderly population. Findings of the study offer insight into obesity and underweight prevalence as well as the relationship between BMI and WHR with chronic medical conditions. More research is also needed to understand the environmental and biological factors which are responsible for the ethnic differences in the population.

## Abbreviations

BMI, Body Mass Index; MICE, multiple imputations by chained equations; MNAR, missing not at random; NHS, National Health Services; TIAs, transient ischemic attacks; WHO, World Health Organization; WHR, waist-hip ratio; WiSE, well-being of Singapore elderly

## Additional file

Additional file 1: Table S1. Factors associated with missing data. Examination of missing data mechanisms which suggests missing not at random (MNAR). (DOCX 17 kb)
