# Supplementary material for: Body mass index, waist-hip ratio and risk of chronic medical condition in the elderly population: results from the Well-being of the Singapore Elderly (WiSE) Study
Source: BMC Geriatr. 2016 Jun 18;16:125. doi: 10.1186/s12877-016-0297-z (PMC4912714; doi:10.1186/s12877-016-0297-z)
Supplement: Additional file 1: Table S1. — Factors associated with missing data. Examination of missing data mechanisms which suggests missing not at random (MNAR). (DOCX 17 kb) [file 12877_2016_297_MOESM1_ESM.docx]

**Supplementary Table S1: Factor associated with missing data**

| **Variable** |  | | | |
| --- | --- | --- | --- | --- |
|  | Odds Ratio | (95% CI) |  | P Value |
|  |  | Lower | Upper |  |
| **Age Group (in years)** |  |  |  |  |
| 75-84 | 2.338 | 1.302 | 4.2 | **0.0045** |
| 85+ | 11.009 | 6.233 | 19.445 | **<.0001** |
| 60-74 | 1 (reference) |  |  |  |
| **Ethnicity** |  |  |  |  |
| Malay | 3.241 | 2.086 | 5.034 | **<.0001** |
| Indian | 1.546 | 0.974 | 2.453 | 0.0646 |
| Others | 0.637 | 0.065 | 6.266 | 0.6991 |
| Chinese | 1 (reference) |  |  |  |
| **Gender** |  |  |  |  |
| Women | 1.687 | 0.915 | 3.108 | 0.0937 |
| Men | 1 (reference) |  |  |  |
| **Marital Status** |  |  |  |  |
| Never Married | 1.453 | 0.43 | 4.912 | 0.5473 |
| Widowed | 1.143 | 0.686 | 1.903 | 0.6083 |
| Divorced/Separated | 1.198 | 0.253 | 5.665 | 0.8195 |
| Married/Cohabiting | 1 (reference) |  |  |  |
| **Education** |  |  |  |  |
| None | 2.011 | 0.737 | 5.489 | 0.1728 |
| Some, but did not complete primary | 0.997 | 0.366 | 2.717 | 0.9953 |
| Completed primary | 1.523 | 0.567 | 4.089 | 0.4043 |
| Completed secondary | 1.571 | 0.566 | 4.363 | 0.386 |
| Completed tertiary | 1 (reference) |  |  |  |
| **Employment Status** |  |  |  |  |
| Unemployed | 0.602 | 0.057 | 6.415 | 0.6745 |
| Homemaker | 4.568 | 1.085 | 19.234 | **0.0384** |
| Retired | 9.537 | 2.503 | 36.335 | **0.001** |
| Paid work (part-time and full-time) | 1 (reference) |  |  |  |
